# A Rare Case of Fractured Posterior Facet of the Talus in Association With Open Medial Subtalar Dislocation

**DOI:** 10.7759/cureus.13224

**Published:** 2021-02-08

**Authors:** Adnan Ahmed, Muthukumar S Balaji, Dilip Kumar Naidu, Hardik L Patel, Vamsi Krishna

**Affiliations:** 1 Department of Orthopaedics, SRM Medical College Hospital and Research Centre, Chennai, IND

**Keywords:** ankle and foot, subtalar dislocation, medial subtalar dislocation, talus fracture

## Abstract

Subtalar or peritalar dislocation is the loss of contact between the articular surface of the talus distally and the calcaneum and navicular. In this paper, a case of open medial type of subtalar dislocation associated with fractured posterior facet of the talus in a 27-year-old man with a history of road traffic accident was reported. Immediate wound irrigation and open reduction under general anesthesia at the emergency room operation theater was successful followed by cast immobilization. At one-year follow-up, the patient was walking and carrying out his daily activities with mild restriction of inversion and eversion movements. Extensive wound debridement followed by immediate reduction and, when required, stabilization are the principal features of management. Open subtalar dislocation is an extremely rare injury and often poses a treatment dilemma. Early debridement and open reduction of the dislocation like in our case can give good functional outcome for an open medial subtalar dislocation at one-year follow-up. Temporary stabilization of dislocation in the form of Kirschner wires maybe needed in some cases only.

## Introduction

Subtalar dislocation is defined as the dislocation of the talar distal articulations at the talonavicular and talocalcaneal joints. In 1811, Judey and Dufaurets first described this injury [1]. Approximately 1-2% of traumatic foot injuries are a result of subtalar fracture-dislocations.

Broca had classified the subtalar joint dislocations (STJDs) into three types: (1) medial, (2) lateral, and (3) posterior dislocation. The principal factor to classify the type of dislocation was the position of the foot with respect to the talus [2].

The most usual type is medial dislocation, also called “acquired clubfoot”, with an incidence of 65-85% of all subtalar dislocations. The next most common is lateral dislocation, or “acquired flat foot”, making up the remaining 15-35% [2].

The stability of the subtalar joint is mainly dependent on ligamentous structures. In a medial dislocation, there is forceful inversion of the foot in a plantar-flexed position, which puts stress on the lateral collateral ligaments, resulting in tear of the talocalcaneal and talonavicular ligaments. In lateral dislocations, the causative mechanism is traumatic eversion of the foot.

The mechanisms of injury, which can result in STJD, include fall from height, road traffic accidents, sports activities, and injuries while running/twisting. Generally, 75% are closed and 25% are open STJDs. Open dislocations usually occur due to high energy mechanisms of injury. The talus is the most commonly injured bone followed subsequently by the ankle, calcaneum, and navicular bone in high energy trauma or twisting. The cuneiforms, cuboid, and metatarsals are rarely injured [3].

## Case presentation

**Figure 1:**
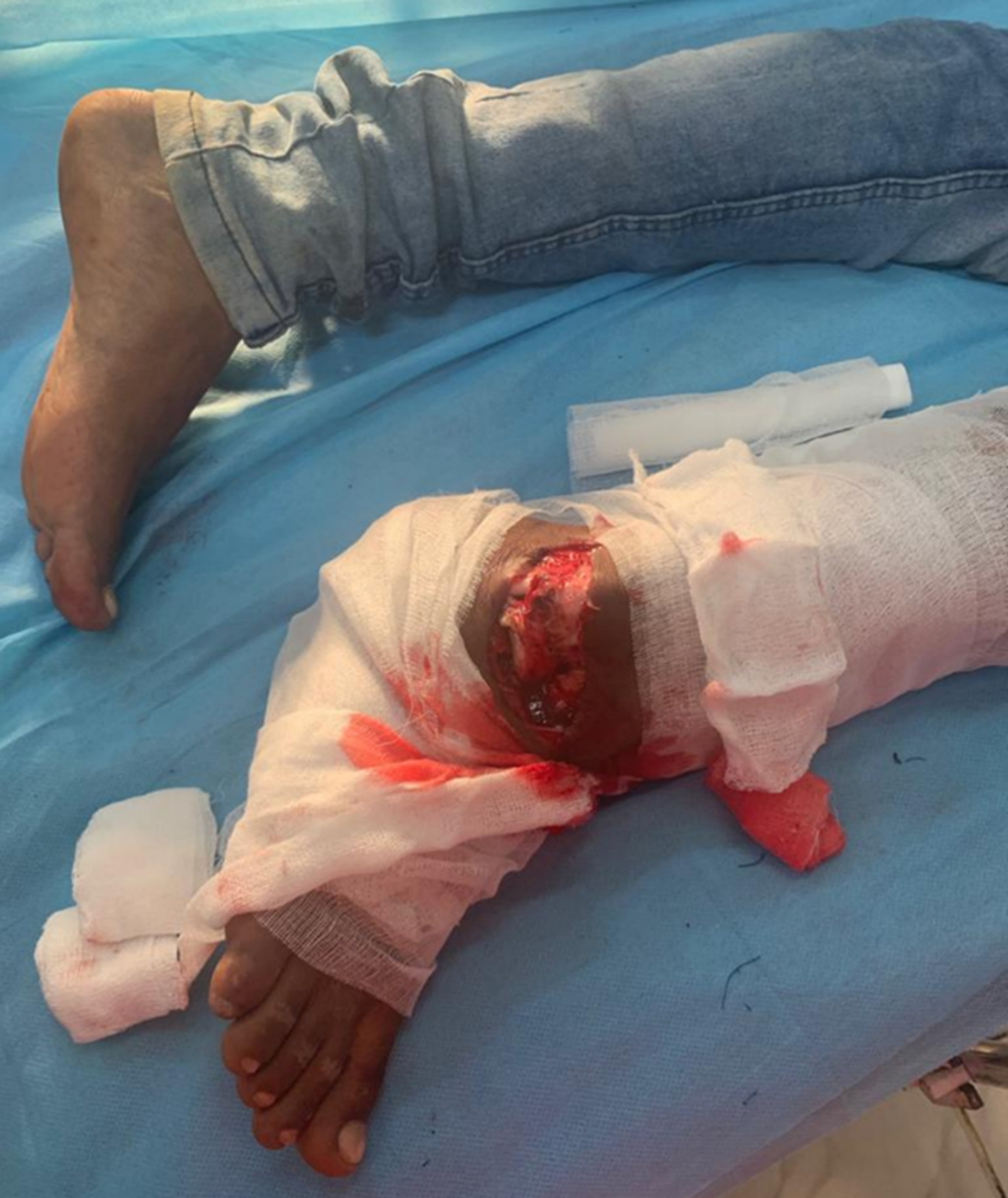
Patient presented in the ER with an injury to his right ankle.

**Figure 2:**
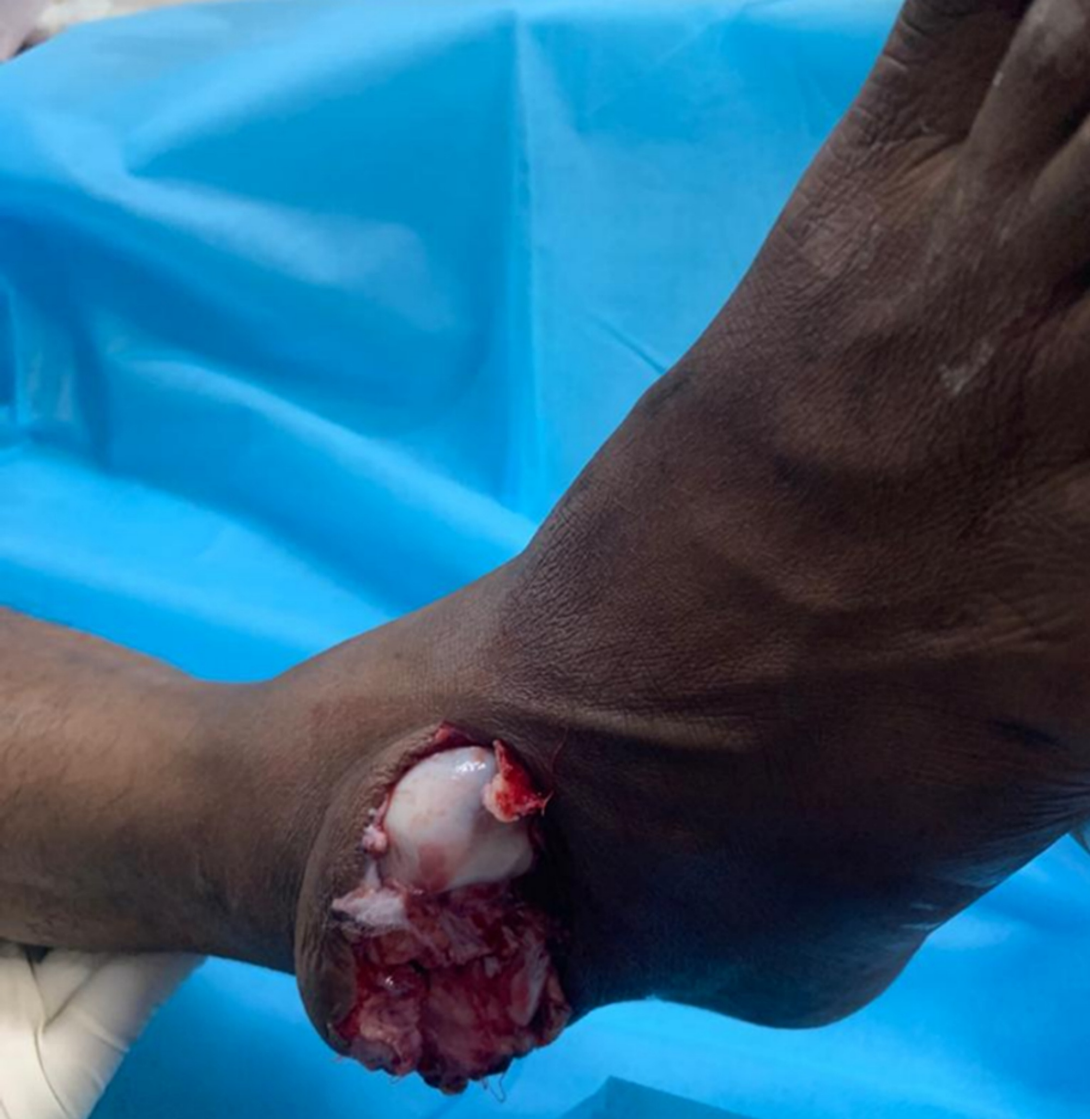
Clinical picture of the right foot showing talus protruding out from the dorsolateral side of the ankle. Foot is in the equinovarus position. The calcaneum is displaced medially.

**Figure 3:**
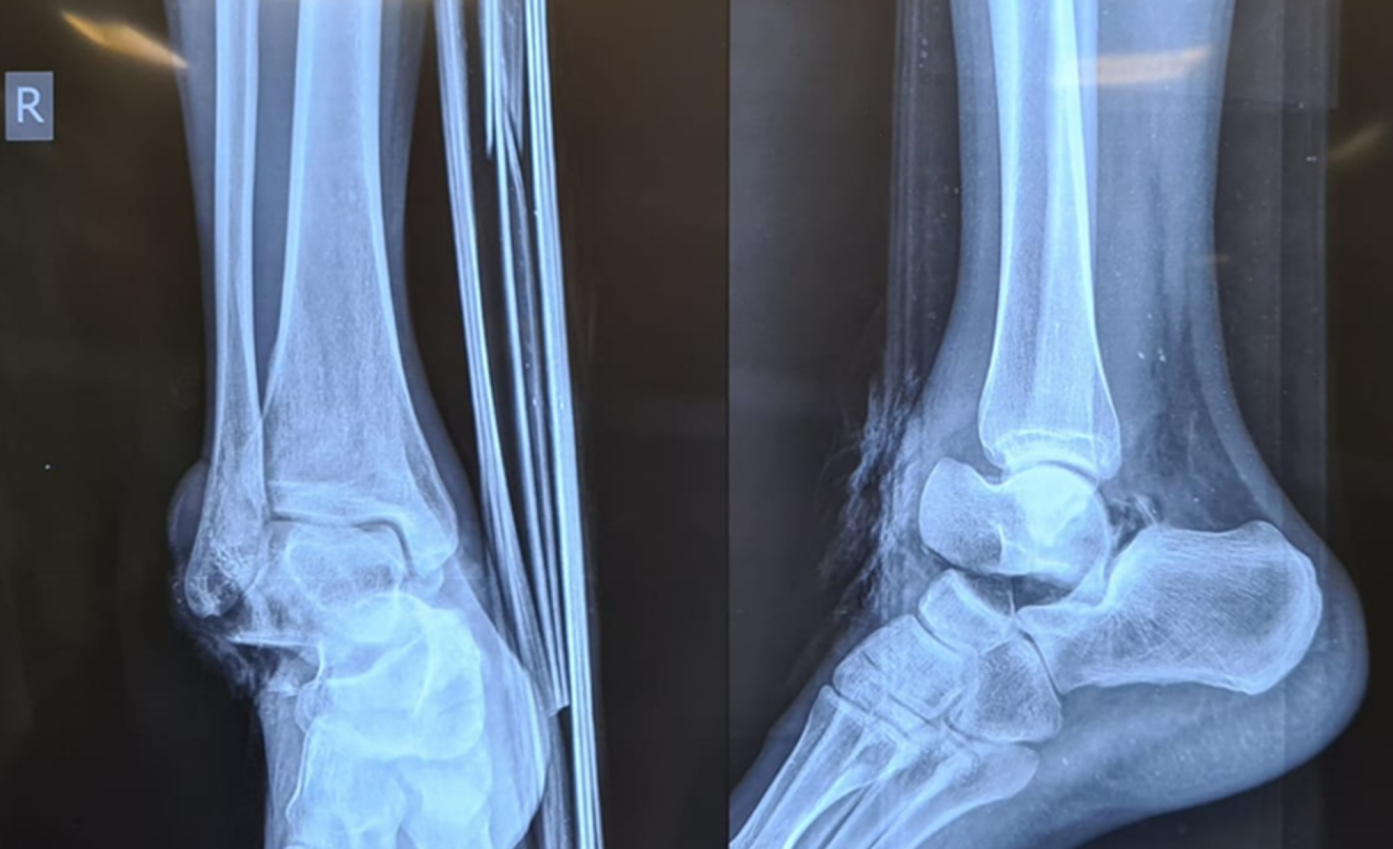
Plain radiograph of the right ankle (anteroposterior and lateral views) showing medial subtalar dislocation. The lateral view shows that the talar head is superior to the navicular.

**Figure 4:**
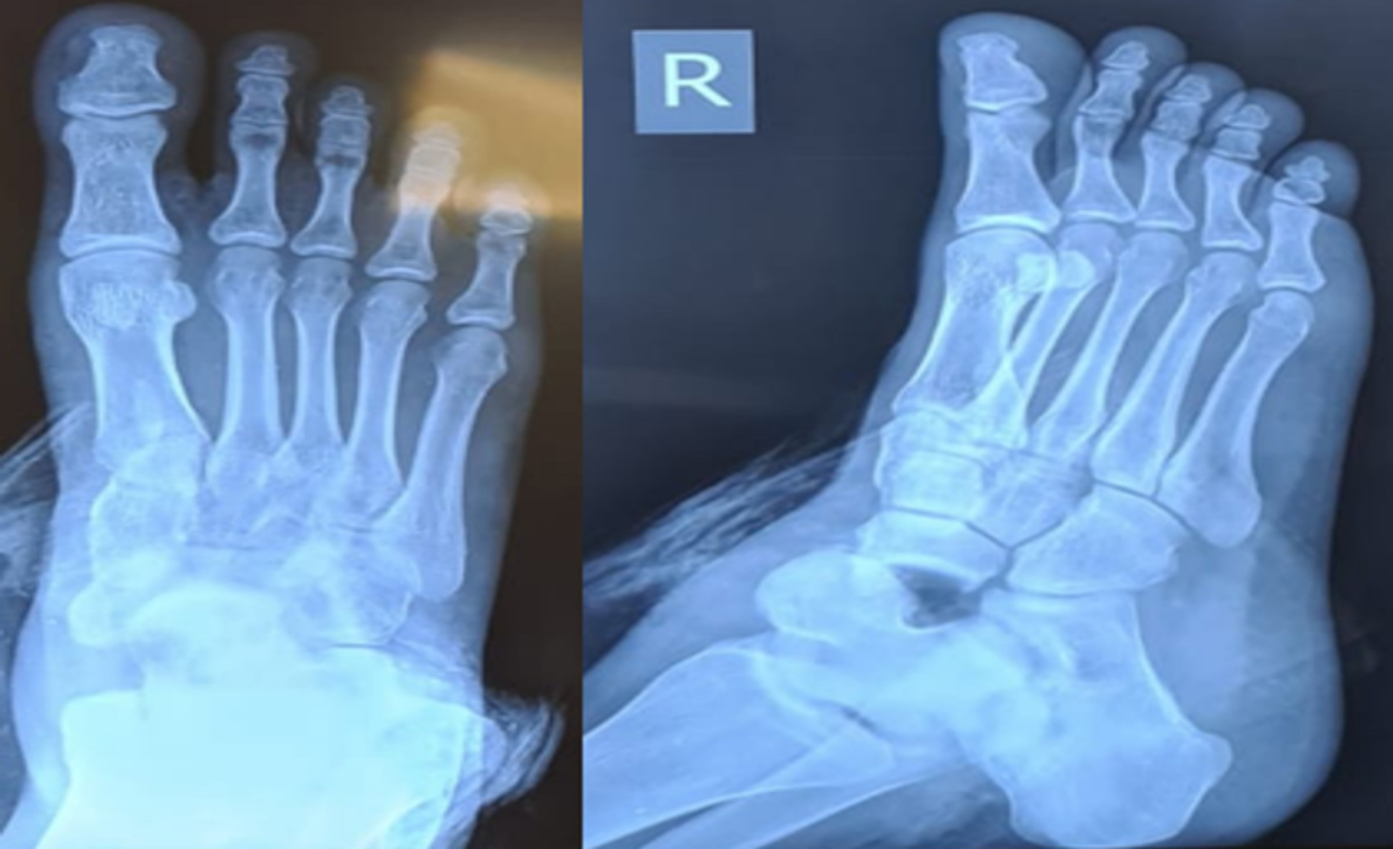
Plain radiographs of the right foot (anteroposterior and oblique views) showing displacement of the talus. The navicular is lying medial to the talar head.

**Figure 5:**
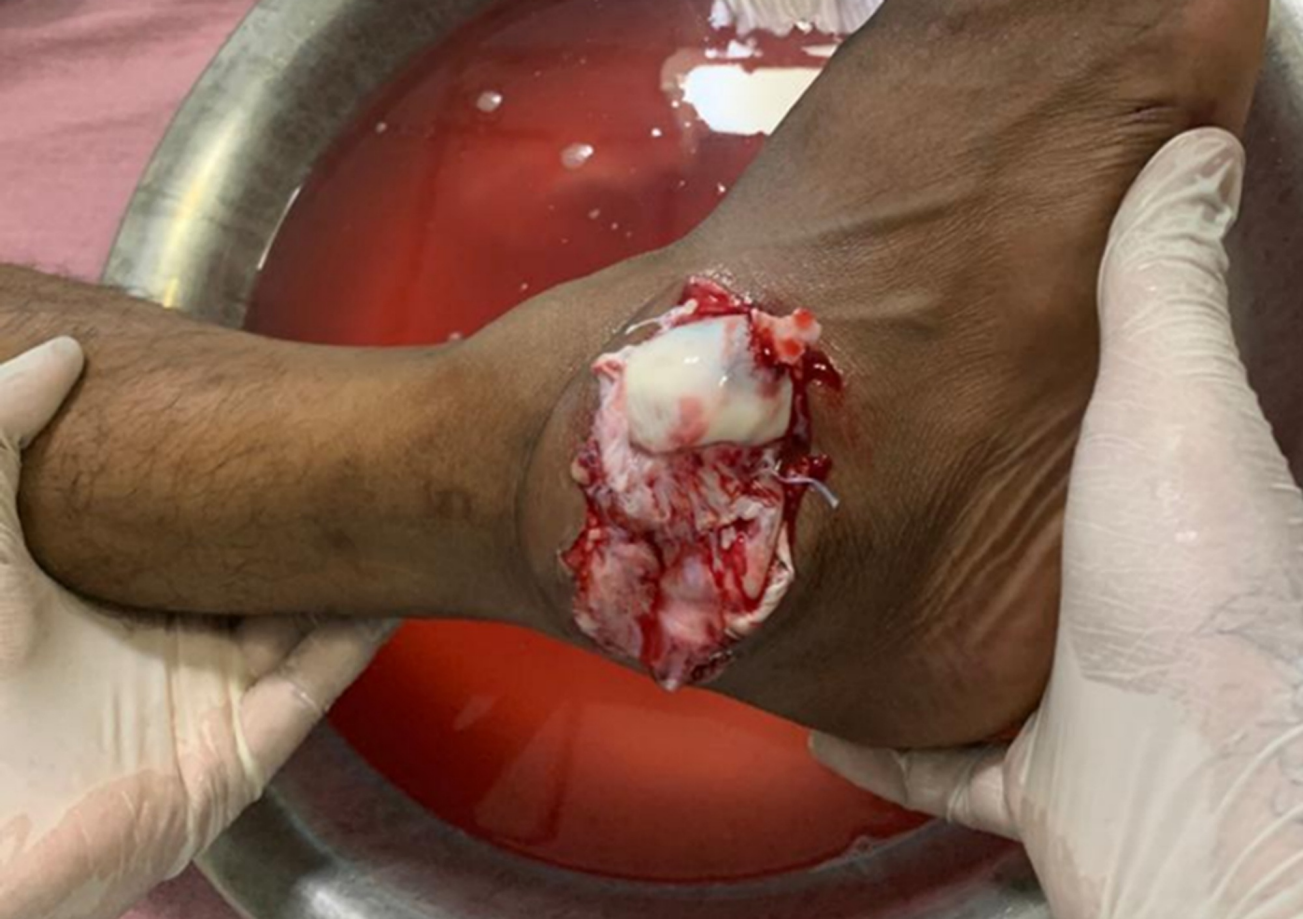
Thorough wound wash given. No tendon or vascular injury was noted.

**Figure 6:**
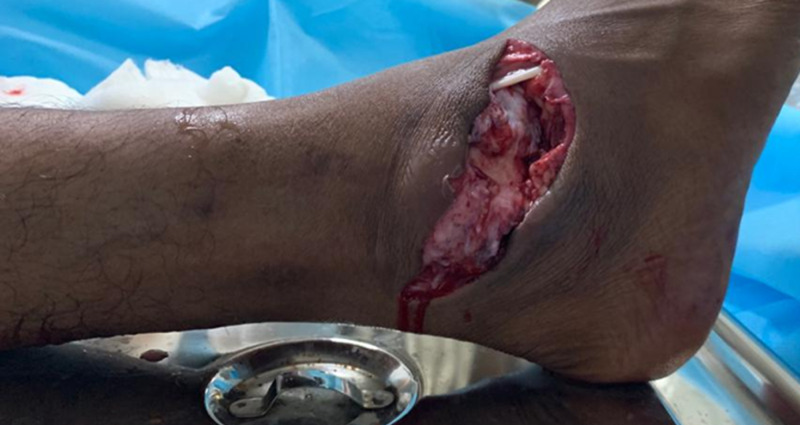
Clinical picture post-reduction. Foot deformity was corrected, and the ankle and subtalar joints were found to be stable. Reduction was achieved with the technique described with knee flexed. Reduction was achieved with a palpable clunk.

**Figure 7:**
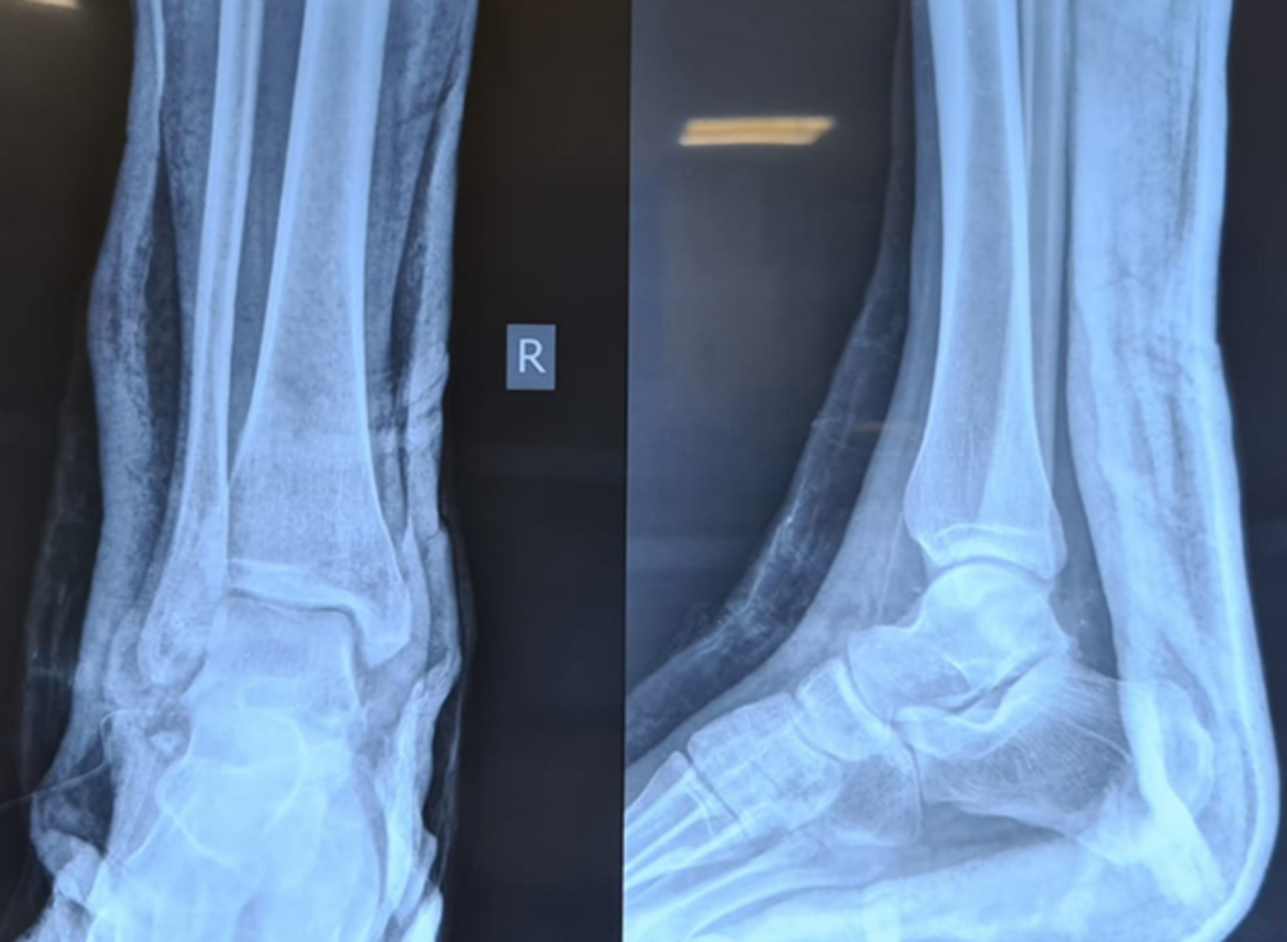
Post-reduction radiograph showing anatomical reduction of the subtalar joint.

**Figure 8:**
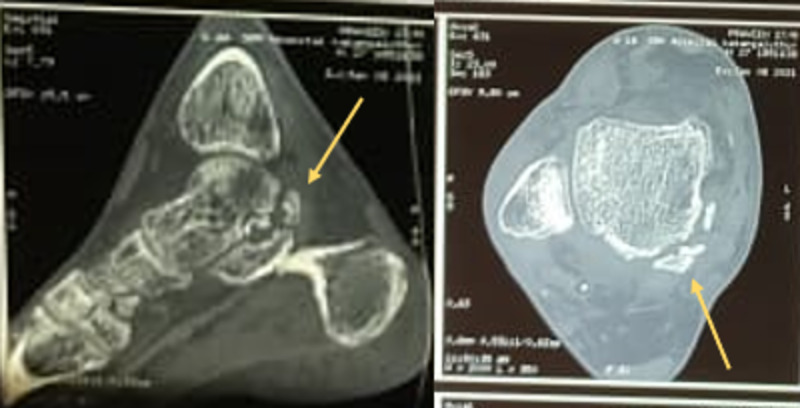
CT scan of the right ankle post-reduction showing fracture of the posterior facet of the talus.

**Figure 9:**
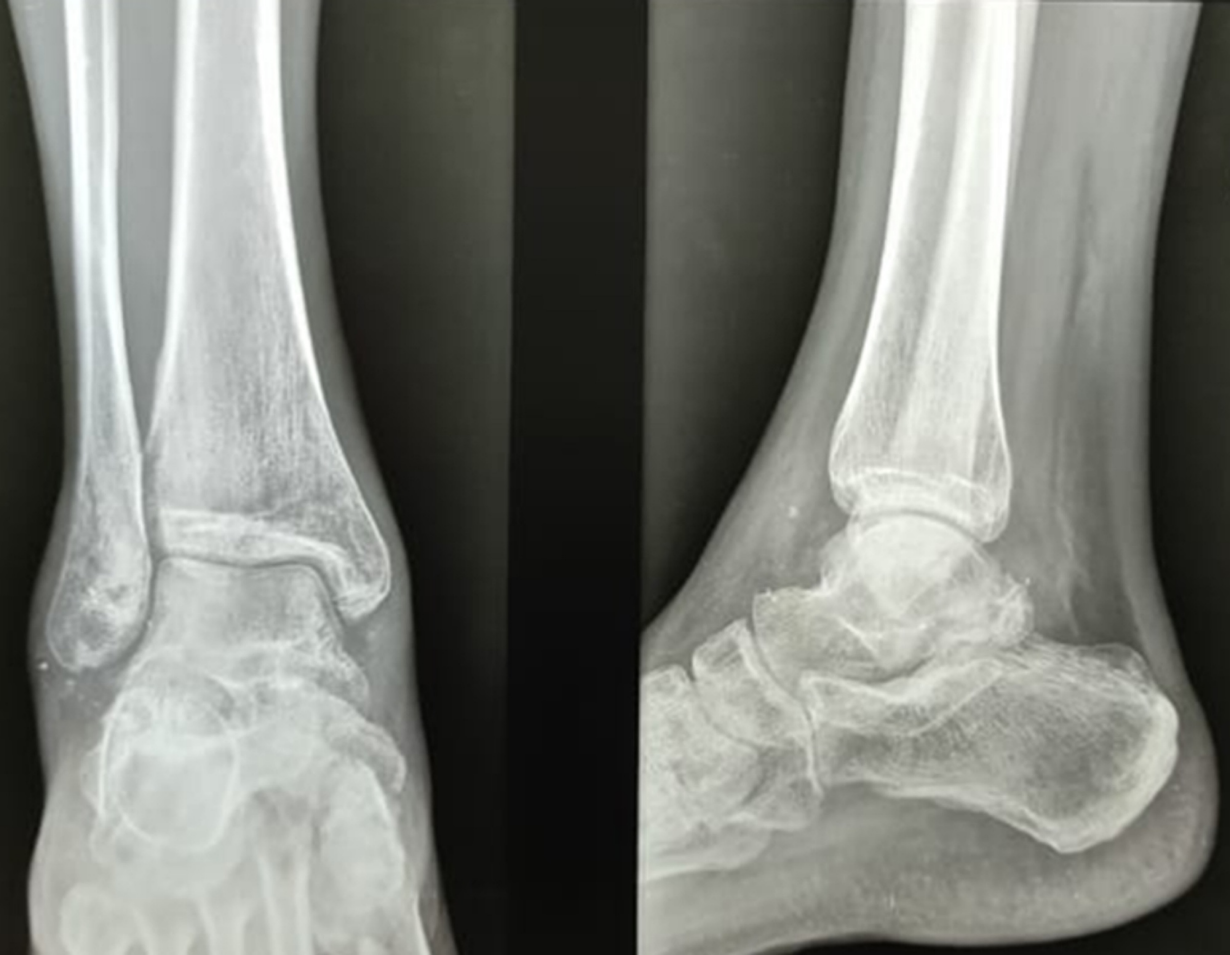
Plain radiographs of right ankle (anteroposterior and lateral views) at one-year post-injury. Hawkins sign is positive, suggestive of good vascularity of the talus.

**Figure 10:**
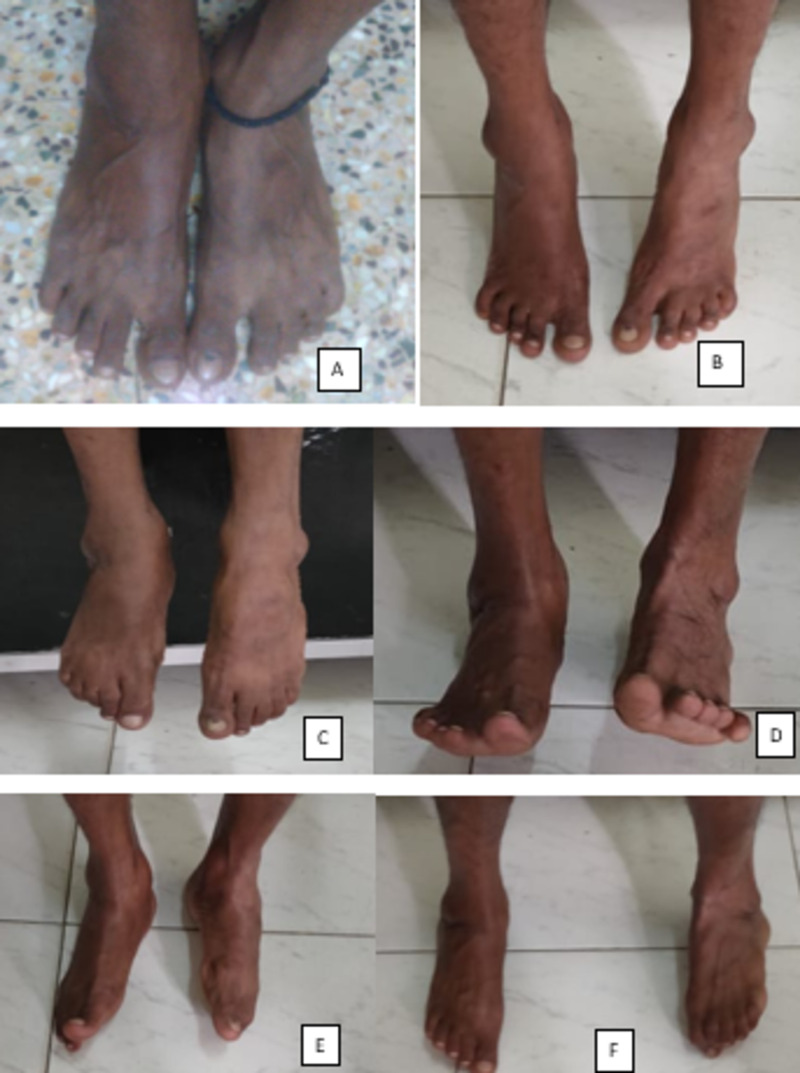
Clinical pictures at one year follow up. (A) Patient standing without pain. (B) Patient able to stand on tiptoes. (C) Plantar flexion. (D) Dorsiflexion. (E) Inversion. (F) Eversion.

A 27-year-old male presented to the emergency room with a history of road traffic accident and sustained trauma to his right ankle (Figure 1). He gave a history of road traffic accident while riding a two-wheeler wherein his right foot was resting on the footpad, and upon impact with another two-wheeler, his foot got twisted inwards. The patient complained of severe pain and was unable to bear weight. On examination, the right foot was in the equinovarus position. There was obvious deformity of the ankle, with bone protruding on the lateral aspect through a 7 x 2 cm laceration (Figure 2). Dorsalis pedis was palpable, and capillary refill and SpO2 were normal. No neurovascular deficit was noted. Radiographs of right ankle were taken and the patient was diagnosed to have open medial subtalar dislocation (Figures 3, 4). Under general anesthesia, thorough wound wash was given. Partial tear of lateral collateral ligament was seen, and no tendon or vascular injury was noted (Figure 5). Reduction was performed with knee flexed with longitudinal traction, inversion, and firm digital pressure over the protruding talus, and a palpable clunk was heard (Figure 6). Post-reduction, ankle and subtalar joints were checked for stability and were found to be stable. Primary skin suturing was performed, and the patient was immobilized in short leg cast. Post-reduction radiographs and CT scan were taken to confirm the reduction and rule out associated fractures (Figure 7). Anatomical reduction was achieved, and CT scan showed associated fracture of the posterior facet of the talus (Figure 8). The patient was planned for ligament repair/reconstruction with Kirschner-wire (K-wire) stabilization, but due to several factors, surgery was deferred and the patient was managed conservatively and was put on non-weight-bearing short leg cast for six weeks (three weeks of non-weight-bearing followed by three weeks of weight-bearing walking) followed by physiotherapy of the ankle and foot. Sutures were removed on post-operative day 12, and wound condition was healthy. Plain radiographs at one year showed no signs of avascular necrosis (AVN) of the talus or arthritic changes in the subtalar joint (Figure 9). At one-year follow-up, the patient was able to perform active ankle motion without pain on walking and with terminal reduction in inversion and eversion movements (Figure 10). The patient was able to perform routine activities without pain with good functional recovery.

 

## Discussion

Peritalar injuries are rare and are rarely missed because of obvious deformity. Low overall incidence, different manifestations, subtle radiographic findings, and distracting injuries can lead to misdiagnosis and late presentation [4]. A detailed history including mechanism of injury along with a thorough physical examination is necessary to come to an accurate diagnosis. In our case, there was an obvious open STJD with laceration.

Subtalar dislocation is noted more often in road traffic accidents than in sports injury because it involves greater velocity of force and higher energy transfer [5]. Lateral STJD is even rarer. Patients with this type of dislocation show severe soft tissue damage [6]. This injury is rare due to strong ligaments connecting the talus and the calcaneum and due to the biomechanical qualities of the ankle [2,7]. In a study conducted by Perugia et al., the AOFAS (American Orthopedic Foot and Ankle Score) scale score showed no statistically significant difference between the medial and lateral STJD at seven-year follow-up [8].

The difference between medial subtalar and medial swivel dislocations is important to understand the mechanism of reduction. Traction and eversion is used to reduce a medial subtalar dislocation, and, on the other hand, traction with lateral rotation of foot is needed to reduce swivel. Possible causes of obstruction to reduction can be advocated to be interposition of the tibialis posterior tendon or impacted fractures of the talus or navicular [9,10].

Closed and open subtalar dislocations may occur in conjunction with fractures of lower extremity. An unusual case of medial subtalar dislocation together with fractured navicular and posterior talus was first reported by Eisenstein et al. [11]. The probable mechanism for the occurrence of posterior facet of talus fractures are from either direct impingement from the posterior tibial plafond or an avulsion of the posterior talofibular ligament [12,13]. In our case, a CT scan was performed, which showed associated posterior facet of talus fracture, which was treated conservatively.

The study conducted by Bibbo et al. showed that the use of CT would generally reveal unsuspected fractures and provide better insight and clarity of the injury. This is necessary in deciding the management plan for acute operative and long-term treatment. Therefore, it was recommended that both radiographs and CT scan are performed for STJD [14]. In our case, CT scan provided valuable information regarding the associated fracture of the talus.

Emergency management in the form of urgent reduction should be done under anesthesia to reduce the occurrence of skin, and nerve or blood vessel complications [10]. The method of reduction depends on the type of dislocation and mechanism involved. Manual traction method usually results in successful reduction, but if manual reduction fails, another technique can be attempted using a 3.5-mm Steinmann pin in the calcaneum. This helps in giving adequate traction, controlling rotation and also stabilizing the talus, and therefore aiding in successful reduction [15]. If there is neglected subtalar dislocation, the gold standard is arthrodesis of the tibia-talus-calcaneum as it ensures satisfactory stability [16]. In our case, since it was open reduction, manual technique was successful.

In a study conducted by Giannoulis et al., which described 439 patients with closed type of pure subtalar dislocation, 85% of patients were treated conservatively and good-to-excellent results were obtained [2]. In a separate study by Fotiadis et al., it was suggested that conservative management is the primary choice, but the long-term results are unpredictable [17]. Rida-Allah et al. described the possible complications to be decreased range of motion (ROM), persistent instability, AVN of the talus (upto 40%) and early degenerative changes (upto 50%) [18].

Due to paucity of cases, the optimal time of immobilization of the ankle and foot remains yet to be decided. A non-weight-bearing below-knee cast is usually kept for three weeks to four months. In the study conducted by Lasanianos et al., early ankle ROM exercises after medial subtalar dislocation appear to achieve better functional results as they help the tendons and ligaments to heal without altering proprioception of the joint [19,20].

In the above case, a below-knee cast was applied for six weeks (three weeks of non-weight-bearing and three weeks of weight-bearing walking) followed by passive and active mobilization after cast removal. This resulted in good functional recovery, with an AOFAS score of 82 out of 100 at one-year follow-up, and the patient was walking pain-free and carrying out all activities of daily living including his vocation without pain. Mild restriction of inversion (10 degrees) and eversion (20 degrees) is present without any adverse effect on his day-to-day activities.

## Conclusions

STJD is an extremely rare injury and often poses a treatment dilemma. Early debridement and open reduction of the dislocation can produce good functional outcomes in an open medial subtalar dislocation at one-year follow-up. Temporary stabilization of dislocation in the form of K-wires maybe needed in some cases only.
